# Differential impacts of parental attention deficit/hyperactivity disorder on early maternal‐infant attachment

**DOI:** 10.1002/jcv2.70029

**Published:** 2025-06-27

**Authors:** Elyse Mark, Brooke S. G. Molina, Michelle A. Wilson, Charles H. Zeanah, Heather M. Joseph

**Affiliations:** ^1^ School of Medicine University of Pittsburgh Pittsburgh Pennsylvania USA; ^2^ Department of Psychiatry School of Medicine University of Pittsburgh Pittsburgh Pennsylvania USA; ^3^ Department of Psychiatry University of Pittsburgh Medical Center Pittsburgh Pennsylvania USA; ^4^ Department of Psychiatry and Neurology School of Medicine Tulane University New Orleans Los Angeles USA

**Keywords:** ADHD, maternal‐infant attachment, maternal‐infant bonding, postpartum depression

## Abstract

**Background:**

Parental attention deficit/hyperactivity disorder (ADHD) is associated with increased postpartum depressive symptoms and impaired daily functioning, potentially impacting early maternal‐infant attachment (MIA).

**Methods:**

78 mothers, half with ADHD, were enrolled during pregnancy or postpartum. Participants completed questionnaires regarding social support, home chaos, postpartum depressive symptoms, and postnatal MIA. Pregnant participants (*n* = 45) also reported antenatal MIA. ANOVA compared mothers without ADHD (*n* = 44), mothers with ADHD (*n* = 21), and mothers with ADHD coparents (infants' fathers; *n* = 13), and Benjamini‐Hochberg correction was applied to account for multiple testing. Multilevel linear regression examined predictors of MIA.

**Results:**

Mothers with ADHD reported greater postpartum depressive symptoms and home chaos than mothers with ADHD coparents or those without parental ADHD; mothers with ADHD coparents reported the poorest overall MIA and quality of attachment and greatest hostility toward their infants (Benjamini‐Hochberg false discovery rate *q* < 0.05). In the final model for MIA, postpartum depressive symptoms (*B* = −0.85, *p* < 0.001) and coparent ADHD (*B* = −4.70, *p* = 0.051) were associated with poorer MIA (*R* ^*2*^ = 0.40, *p* < 0.001). When examining MIA subscales, postpartum depressive symptoms were negatively correlated with subscales measuring quality of attachment and absence of hostility but not pleasure in mother‐child interaction. When antenatal attachment was included, it (*B* = 0.65, *p* = 0.002), along with postpartum depressive symptoms (*B* = −0.60, *p* = 0.032), predicted postnatal MIA (*R* ^*2*^ = 0.50, *p* < 0.001).

**Conclusions:**

Given parental ADHD (mother or father) was associated with increased maternal postpartum depressive symptoms and less optimal MIA in this study, additional support for families with ADHD in the perinatal period may improve maternal and infant mental health outcomes. Future work incorporating observational data and additional investigation of the coparenting relationship is needed to further clarify determinants of MIA in early childhood.

## Key Points

- Postpartum depressive symptoms have been reliably associated with less optimal maternal‐infant attachment (MIA), specifically lower quality of attachment and greater infant‐directed hostility.
- Mothers diagnosed with attention‐deficit/hyperactivity disorder (ADHD), as well as those with coparents with ADHD, report poorer attachment to their infants compared to mothers in non‐ADHD parenting pairs.
- Regression analyses indicate that both maternal postpartum depressive symptoms and presence of a coparent with ADHD negatively impact MIA in the neonatal period.
- *How* and *why* parental ADHD, in either the birthing or non‐birthing parent, negatively affects early parental bonding remains poorly understood, warranting future investigation of symptom‐specific peripartum impairments.
- Providing expectant parents with ADHD with preventative interventions focused on ADHD symptom management may improve MIA and family functioning.

## INTRODUCTION

Women with attention deficit/hyperactivity disorder (ADHD) are significantly more likely to develop postpartum depression than those without ADHD, even after accounting for known contributors such as age, other psychiatric history, and marital status (Andersson et al., 2023). Though specific mechanisms linking ADHD with disordered peripartum mood are unclear, baseline impairments in attention and executive function in adults with ADHD may be exacerbated by the demanding transition to parenthood and worsen baseline emotional dysregulation. Even among those lacking ADHD diagnoses, elevated ADHD symptoms during pregnancy have been linked to increased functional impairment in daily life and interpersonal relationships (Eddy et al., 2019) and frequency of infant maltreatment (Oliveira et al., 2022). Moreover, the negative effects of ADHD on postpartum health and functioning may not be confined to the diagnosed adult, with recent literature revealing associations between parent or coparent ADHD and parenting distress in the first year of life (Joseph et al., 2022). While adult ADHD, postpartum mood, and parenting experiences appear closely connected, the intersection of parental ADHD and maternal‐infant attachment (MIA) in the postpartum period remains unexplored.

### Maternal‐infant attachment

Maternal‐infant attachment refers to the emotional bond between mother and baby, which evolves throughout the peripartum period and is believed to lay the groundwork for the child's development of emotional regulation and social relationships through adulthood (Girme et al., 2021). Strong MIA (also called “mother‐infant bonding,” “maternal‐infant bonding,” or “maternal bonding”) has been linked to improved social‐affective, motor, and language outcomes in infancy (Le Bas et al., 2022; Sierau et al., 2016) and fewer behavioral issues through childhood and adolescence (Reyes et al., 2021). Impaired MIA manifests as decreased maternal warmth and responsiveness, with postpartum depression consistently identified as a key contributing factor (Behrendt et al., 2016; Dubber et al., 2015; Farre‐Sender et al., 2018; Makeen et al., 2022). Even subclinical postpartum depressive symptoms have been associated with poorer MIA (Behrendt et al., 2016; Moehler et al., 2006), particularly when concurrent with postpartum anxiety (Tietz et al., 2014). A substantial literature has explored postpartum depression and the early relationship between mother and infant, finding depressive symptomatology related to decreased maternal engagement and structuring behaviors (Hakanen et al., 2019), as well as increased irritability and infant‐directed hostility (Lovejoy et al., 2000; Mavrogiorgou et al., 2022). Cognitive distortions experienced by mothers with postpartum depression may underlie dysfunctional mother‐child interaction, with recent research highlighting negative associations between rumination and parenting characteristics, such as maternal sensitivity and engagement (Stein et al., 2012; Tester‐Jones et al., 2017).

Several other maternal psychiatric and sociodemographic factors have been linked to MIA. Fewer studies have considered peripartum anxiety as an isolated determinant of MIA, and while most that have reported a direct correlation between maternal anxiety and MIA, some found that this association was not significant after controlling for depression (Dubber et al., 2015; Lehnig et al., 2019; Tietz et al., 2014). Other mental health factors related to impaired maternal bonding include lifetime history of depression or anxiety (Petri et al., 2018) and history of abuse in childhood (Chau et al., 2023; Lehnig et al., 2019). While studies have linked marriage (Petri et al., 2018) and social support (Delavari et al., 2018; Doyle et al., 2023) to improved MIA, findings regarding other social and demographic characteristics are mixed. For example, some have reported stronger MIA among younger women (Bicking et al., 2014) and other studies have reported stronger MIA among older women (Doyle et al., 2023); the role of educational attainment remains similarly unclear (Bicking et al., 2014; Moehler et al., 2006).

Infant temperament, meaning individual differences in affectivity and reactivity emerging early in life, has been studied as a child‐level factor that may impact MIA. The directionality of this relationship is poorly understood, however. While Takács et al. (2020) found that MIA in the immediate postpartum period predicted early infant temperament, they and others have also shown that by six to 9 months postpartum, child temperament seems to drive maternally reported attachment scores (Nolvi et al., 2016; Takács et al., 2020).

### Attachment in ADHD

While little is known about the specific contributions of parental ADHD to early parent‐to‐infant attachment, many researchers have investigated attachment representations among children and adults with ADHD outside of the perinatal period. Children with ADHD show increased incidence of ambivalent and disorganized attachment (Dekkers et al., 2021), and, conversely, infants (Pinto et al., 2006) and children (Bohlin et al., 2012; Scholtens et al., 2014) with disorganized attachment exhibit more ADHD‐related symptoms during childhood. The relation between attachment and ADHD symptoms appears to persist beyond adolescence, as adults with ADHD are more likely to demonstrate insecure attachment styles than the general population (Storebø et al., 2016). Moreover, in a sample of 73 adults with ADHD, Edel et al. (2015) found significant associations between adults' recollections of their own parents' ADHD symptoms experienced as children and their own current emotional dysregulation, as well as ongoing attachment disturbances in romantic relationships. Potential negative impacts of disrupted MIA are particularly important for the offspring of parents with ADHD, who are already at elevated risk for disruptive behaviors and poor emotion regulation due to the profound heritability of ADHD (recently estimated at 74%) (Faraone & Larsson, 2019).

There are several ways parental ADHD may impair MIA. As previously discussed, mothers with ADHD are at higher lifetime risk of depression (Choi et al., 2022), including during the postpartum period (Andersson et al., 2023). Poorer social support, particularly coparent support for the daily activities of parenting, may also negatively affect MIA. Several studies have demonstrated that individuals with ADHD or with partners with ADHD experience more frequent and severe conflict in their romantic relationships (Bruner et al., 2015; Kahveci & Tutarel, 2022), with non‐ADHD adults commonly reporting feeling “overwhelmed” or “burdened” by compensation for functional deficits in their partners' attention and executive function (Zeides Taubin & Maeir, 2023). Higher baseline conflict in partnerships with adult ADHD, along with increased compensatory demands on the non‐ADHD parent during the newborn period, may contribute to greater perceived household chaos and disrupt early parent‐infant bonding. In addition, studies suggest offspring who later develop ADHD (symptoms or diagnosis) are more likely to show negative emotionality in infancy and toddlerhood (Joseph et al., 2023), which may further intensify household stress and strain MIA.

### The current study

Families with parental ADHD may be particularly vulnerable to peripartum mood disorders, parenting stress, and impaired MIA, with important implications for parental mental health and infant development. The present study investigates the role of parent and coparent ADHD in MIA among a sample of mothers with ADHD, mothers whose coparents have ADHD, and those in parenting pairs without ADHD. We test the hypotheses that parent ADHD (mother or father) is associated with impaired MIA, and that maternal postpartum depressive symptoms, poor social support, and increased home chaos are associated with both parental ADHD and decreased MIA.

## MATERIALS AND METHODS

### Participants

Participants were mothers in southwestern Pennsylvania recruited during pregnancy or at the time of delivery for participation in the New(born) Pittsburgh ADHD Risk in Infancy Study (PARIS). The parent study enrolled 78 biological mother‐father pairs, half with and half without parental ADHD, along with their newborns, to longitudinally examine early predictors of childhood (offspring) ADHD through parent interviews, parent surveys, and computer‐based attention and interaction tasks administered to parents and children across the first 18 months of child life. Of the families with ADHD (*n* = 34), at least one parent met DSM‐5 criteria for childhood ADHD with persistence into adulthood; 21 included a mother with ADHD, 12 included a father with ADHD, and one included two parents with ADHD. Families were excluded if their infant was born earlier than 37 weeks of gestation, with low birth weight (<5lbs 7oz), or with perinatal substance exposure as determined by both mother and father report. The present study includes all 78 mothers from the parent study and focuses on maternal mental health in the first month postpartum, as captured through self‐report measures. Maternal characteristics are shown in Table 1.

**TABLE 1:** Maternal characteristics.

	All (*N* = 78)	Parental ADHD^a^ *(n = 34)*	No parental ADHD *(n = 44)*
	*M* (SD)	*M* (SD)	*M* (SD)
Maternal age (years)	32.3 (3.9)	33.1 (3.5)	31.6 (4.0)

	*n* (%)	*n* (%)	*n* (%)
Maternal race			
Asian	3 (3.8)	‐	3 (6.8)
Black or African American	3 (3.8)	1 (2.9)	2 (4.5)
White or European American	71 (91.0)	32 (94.1)	39 (88.6)
More than one race	1 (1.3)	1 (2.9)	‐
Maternal Ethnicity			
Hispanic or Latina	3 (3.8)	2 (5.9)	1 (2.3)
Middle Eastern or North African	3 (3.8)	1 (2.9)	2 (4.5)
Not Hispanic/Latina or Middle Eastern/North African	72 (92.3)	31 (91.2)	41 (93.2)
Highest parental education			
High school or GED	1 (1.3)	‐	1 (2.3)
Technical/Secretarial school	2 (2.6)	‐	2 (4.5)
Partial college (>1 year)	6 (7.7)	4 (11.8)	2 (4.5)
Associate or 2‐year degree	3 (3.8)	1 (2.9)	2 (4.5)
College or university graduate	26 (33.3)	13 (38.2)	13 (29.5)
Graduate or professional training	40 (51.3)	16 (47.1)	24 (54.5)
Maternal lifetime mood disorder^b^	30 (38.5)	16 (47.1)	14 (31.8)
Maternal ADHD diagnosis	21 (26.9)	21 (61.8)	‐

^a^ Mother or father met criteria for persistent ADHD into adulthood on the CAADID.

^b^ History of Major Depressive Disorder, Bipolar I or II Disorders, Generalized Anxiety Disorder, Panic Disorder, or Social Anxiety Disorder.

### Procedures

Master's or doctoral‐level clinicians administered the Structured Clinical Interview for DSM‐5 Disorders (First et al., 2015) to assess lifetime history of mood and anxiety disorders^1^ and the semi‐structured Conners Adult ADHD Diagnostic Interview for the DSM‐5 (CAADID; Epstein et al., 2001) to assess ADHD symptoms in childhood and adulthood. These assessments were conducted with all mothers and fathers in the parent study on enrollment, and parents were identified as having ADHD (yes, no) if they met DSM‐5 criteria for any presentation: 6+ symptoms in childhood and 5+ symptoms in adulthood with impairment across the lifespan. Maternal participants completed questionnaires on their sociodemographic and childbearing histories, baseline home chaos, and baseline social support remotely using Qualtrics (Provo, UT), on enrollment. Self‐report measures on postpartum mental health and attachment were completed by mothers remotely at approximately 4 weeks postpartum. All participants were compensated for their time and the study protocol was approved by the University of Pittsburgh IRB.

### Survey measures

#### Maternal postpartum depressive symptoms

The Edinburgh Postnatal Depression Scale (EPDS; Cox et al., 1987) is a 10‐item scale measuring depressive symptoms in the postpartum period, with each question rated from 0 to 3 and total scores between 0 and 30. Due to the inability to monitor scores in real‐time, this study omitted the final item on suicidality (EPDS‐9); however, a recent meta‐analysis by Qiu et al. (2023) indicated a greater than 99% correlation between the EPDS‐9 and the full EPDS, supporting use of this modified scale to measure depressive symptoms in the present study.

#### Home chaos

The Confusion, Hubbub, and Order Scale (CHAOS; Matheny et al., 1995) is a 15‐item scale designed to measure home chaos. Mothers rated each item (e.g., “It's a real zoo in our home”) on a scale from 1 (Very much like your own home) to 4 (Not at all like your own home). Total scores range from 15 to 60, with higher scores indicating more chaotic homes.

#### Maternal social support index

The Maternal Social Support Index (MSSI; Pascoe et al., 1981) is a 21‐item scale detailing different household and parenting activities, with total scores ranging from 1 to 19. Questions ask about the division of household labor, frequency and quality of contact with relatives and community members, and availability of transportation.

#### Maternal‐infant attachment

The Maternal Postnatal Attachment Scale (MPAS; Condon & Corkindale, 1998) is a 19‐item self‐report questionnaire that measures MIA in the postpartum period. Items are rated on a scale of 1‐5, with higher scores reflecting stronger attachment (Condon, 2015b) and total scores ranging from 19 to 95. The measure includes three subscales: Quality of Attachment (range 9–45), Absence of Hostility (range 5–25), and Pleasure in Interaction (range 5–25). The Quality of Attachment scale measures maternal warmth and affection toward the infant, as well as feelings of parenting competence (e.g. “Over the last 2 weeks I would describe my feelings for the baby as: 1. Dislike—5. Intense affection”). The Absence of Hostility scale assesses how burdened or resentful the respondent may feel toward the infant (e.g. “When I am caring for the baby, I get feelings of annoyance or irritation: 1. Very frequently—5. Never”). Finally, the Pleasure in Interaction scale examines how much the respondent values and enjoys spending time with the infant (e.g. “When I have to leave the baby: 1. I usually feel rather sad (or it's difficult to leave)—5. I usually feel rather relieved (and it's easy to leave)”).

The Maternal Antenatal Attachment Scale (MAAS; Condon & Corkindale, 1997; Condon, 2015a) is also a 19‐item self‐report questionnaire and measures MIA during pregnancy. Questions explore maternal interest in and preoccupation with pregnancy as well as the respondent's emotions toward the growing infant. Similarly to the MPAS, each item is rated 1‐5, with total scores ranging from 19 to 95. Although the MAAS comprises two subscales (Quality of Attachment and Intensity of Preoccupation), the present study used only the summative score to measure total antenatal attachment among the subset of participants enrolled in pregnancy.

### Analysis

The primary outcome of interest was MIA in early infancy (*M* = 4.12, SD = 2.11 weeks postpartum) as measured by total MPAS score and the MPAS Pleasure, Quality, and Absence of Hostility subscales. Psychiatric predictors included parental ADHD (assessed via CAADID symptom count in bivariate and mean comparisons, and as a binary diagnosis in regression analyses), maternal postpartum depressive symptoms, and maternal history of mood or anxiety disorder; environmental variables were maternal report of social support and home chaos. Intercorrelations (Table 2) and descriptive statistics were calculated, and group differences in mean predictors and outcome variables compared using ANOVA between mothers with ADHD, mothers coparenting with fathers with ADHD, and mothers in non‐ADHD parenting pairs (Table 3). Given multiple comparisons, we assessed statistical significance using a Benjamini‐Hochberg false discovery rate (FDR) of *q* < 0.05 (Benjamini & Hochberg, 1995).

**TABLE 2:** Intercorrelations.

		1	2	3	4	5	6	7	8	9
1	Maternal age in years	‐‐								
2	First child^a^ ^,^ ^b^	−0.227*	‐‐							
3	Maternal lifetime mood disorder^a^	0.083	0.085	‐‐						
4	Maternal postpartum depressive symptoms (EPDS)	−0.152	0.013	0.183	‐‐					
5	Social support (MSSI)	0.038	−0.181	−0.050	−0.031	‐‐				
6	Home chaos (CHAOS)	0.282*	−0.303**	0.138	0.257*	0.035	‐‐			
7	MPAS sum	−0.065	0.112	−0.224*	−0.531***	−0.045	−0.320**	‐‐		
8	MAAS sum^c^	−0.331*	0.261	−0.094	0.023	0.045	−0.378*	0.567***	‐‐	
9	Maternal ADHD symptoms^d^	0.133	0.067	0.224*	0.249*	0.039	0.367***	−0.049	−0.021	
10	Paternal ADHD symptoms^d^	0.040	−0.020	0.126	0.187	−0.150	0.271*	−0.278*	−0.414**	−0.078

*Note*: Bolded text indicates statistical significance with *p* < 0.05.

^a^ Spearman correlations calculated for dichotomous variables.

^b^ Dichotomous variable equal to 1 if the participant's current infant, for whom they were enrolled in the study, is her first child; equal to 0 otherwise.

^c^ Antenatal attachment score (MAAS) available for participants enrolled in pregnancy (*n* = 45).

^d^ ADHD symptom count derived from maternal and paternal CAADID testing.

*p < 0.05; **p < 0.01; ***p < 0.001.

**TABLE 3:** Comparison between mothers with ADHD, mothers with coparent (father) ADHD, and mothers in non‐ADHD parenting pairs.

	*M* (SD)	Range	*M* (SD)			*p*	η^2^
			Mother ADHD (*n* = 21)	Father ADHD (*n* = 13)	No ADHD (*n* = 44)		
Maternal postpartum depressive symptoms (EPDS)	5.46 (4.96)	0–20	7.38 (4.98)	6.92 (5.94)	4.11 (4.28)	**0.021**	**0.098**
Social support (MSSI)	14.06 (2.00)	10–18	14.52 (1.57)	13.08 (1.98)	14.14 (2.12)	0.113	0.057
Home chaos (CHAOS)	23.59 (6.54)	15–41	26.95 (6.26)	24.46 (8.50)	21.73 (5.37)	**0.008**	**0.122**
ADHD symptoms^a^	3.95 (5.49)	0–17	12.33 (2.65)	1.54 (2.26)	0.66 (1.43)	**<0.001**	**0.875**
MPAS							
Pleasure in interaction subscale	21.38 (21.38)	9–25	21.33 (3.10)	20.08 (3.84)	21.80 (2.56)	0.187	0.044
Absence of hostility subscale	20.49 (2.77)	14–25	20.44 (2.26)	18.54 (2.52)	21.09 (2.84)	**0.012**	**0.111**
Quality of attachment subscale	40.87 (4.69)	23–45	40.15 (5.50)	38.22 (5.30)	41.99 (3.72)	**0.026**	**0.093**
Total score	82.74 (8.89)	51–95	81.93 (8.80)	76.84 (10.10)	84.88 (7.84)	**0.013**	**0.110**
MAAS^b^ total score	73.93 (7.54)	56–85	73.82 (7.36)	65.71 (7.76)	76.39 (5.97)	**0.003**	**0.247**

*Note*: The *p‐*values presented above are unadjusted. Bolded text indicates statistical significance given Benjamini‐Hochberg FDR of *q* < 0.05.

^a^ Number of ADHD symptoms from CAADID.

^b^ Completed only by participants enrolled in pregnancy (*n* = 45).

Multilevel linear regression was used to analyze the effects of psychiatric and environmental variables on MIA (Table 4). The base model included demographic covariates only (maternal age in years and educational attainment), the parental ADHD model also assessed the additional effect of any parental ADHD diagnosis (mother or father), the gendered ADHD model specified sex of the parent with ADHD, and the full model included the demographic covariates, parental ADHD by parent sex, as well as the remaining maternal psychiatric (postpartum depressive symptoms, lifetime mood or anxiety disorder history) and environmental factors (home chaos) that were correlated with postpartum MIA in the bivariate analysis. The full regression model was also used to predict subscales of postpartum MIA. Secondary analysis on a subset of participants who were enrolled during pregnancy (*n* = 45) added antenatal MIA (MAAS total score) into the model (available in Table S1). All statistical analyses were conducted in SPSS 27 (SPSS, Inc. Chicago, IL) using a threshold for statistical significance of *α* < 0.05 unless otherwise specified.

**TABLE 4:** Multilevel models of postnatal maternal‐infant attachment (*N* = 78).

	Base model *R* ^*2*^ = 0.014 (*p* = 0.589)		Parental ADHD model *R* ^*2*^ = 0.084 (*p* = 0.089)		Gendered ADHD model *R* ^*2*^ = 0.12 (*p =* 0.050)		Full model *R* ^*2*^ = 0.40 (*p <* 0.001)	
	B (SE)	*p*	B (SE)	*p*	B (SE)	*p*	B (SE)	*p*
Maternal age	−0.20 (0.27)	0.456	−0.08 (0.27)	0.754	−0.13 (0.27)	0.627	−0.24 (0.24)	0.327
Parental education	0.80 (0.93)	0.392	0.73 (0.90)	0.420	0.78 (0.89)	0.382	0.03 (0.76)	0.974
Any parental ADHD			−4.79 (2.02)*	**0.020**				
Mother ADHD					−2.72 (2.32)	0.246	1.94 (2.15)	0.369
Father ADHD					−7.98 (2.71)**	**0.004**	−4.70 (2.37)	0.051
Mother postpartum depressive symptoms							−0.84 (0.19)***	**<0.001**
Mother lifetime mood disorder							−0.84 (0.19)	0.193
Home chaos							−0.24 (0.14)	0.105
Constant	83.55 (9.79)***	<0.001	82.31 (9.51)***	<0.001	83.36 (9.41)***	<0.001	101.48 (8.59)***	<0.001

*Note*: Bolded text indicates statistical significance with *p* < 0.05.

*p < 0.05, **p < 0.01, ***p < 0.001.

## RESULTS

Average age of the mothers was 32.26 years, SD = 3.86, enrolled in pregnancy (*n* = 45) or postpartum (*n* = 33), and most identified as white and highly educated (Table 1). Prevalence of prior maternal mood or anxiety disorder was higher among participants in dyads with ADHD than those without (47% vs. 32%), although the difference did not reach statistical significance (*X* ^*2*^ = 1.88, *p* = 0.17).

Bivariate analysis (Table 2) indicated postnatal MIA was negatively associated with prior maternal mood or anxiety disorder (*r = ‐*0.22), home chaos (*r* = −0.32), maternal postpartum depressive symptoms (*r* = −0.24), and paternal ADHD symptom count (*r* = −0.28) (*p* < 0.05). Among participants enrolled in pregnancy, antenatal MIA was moderately positively correlated with postnatal attachment (*r* = 0.57, *p* < 0.001) and moderately negatively correlated with paternal ADHD symptoms (*r* = −0.41) (*p* < 0.01). Higher home chaos was associated with both maternal ADHD symptoms (*r* = 0.37) and paternal ADHD symptoms (*r* = 0.27), as well as incidence of prior maternal mood or anxiety disorder (*r =* −0.22) and maternal postpartum depressive symptoms (*r* = 0.25) (*p* < 0.05).

Comparison between participants with ADHD, those coparenting with fathers with ADHD, and those without parental ADHD revealed several differences in both predictor and outcome variables reaching significance at a Benjamini‐Hochberg FDR of *q <* 0.05 as follows (Table 3). Mothers with ADHD reported the highest home chaos and postpartum depressive symptoms. Regarding postnatal MIA, both overall and across subscales measuring Absence of Hostility and Quality of Attachment, mothers in non‐ADHD pairs reported the strongest MIA and those coparenting with fathers with ADHD the weakest.

Multilevel modeling demonstrated that ADHD in either parent was negatively associated with postnatal MIA when accounting for maternal age and education (Table 4, *p* < 0.05). Moreover, differentiation by sex of parent diagnosed with ADHD showed that while paternal ADHD was significantly negatively associated with MIA (*p* < 0.01), effects of maternal ADHD did not reach statistical significance. Addition of environmental variables and other maternal psychiatric factors in the full model substantially improved model explanatory power (*R* ^*2*^ = 0.40, *p* < 0.001), with results indicating that only maternal postpartum depressive symptoms were significantly associated with poorer postnatal MIA, though effects of paternal ADHD approached statistical significance (*p* = 0.051).

The final models for the MIA subscales accounted for 25%–34% of variance in Pleasure in Interaction (*R* ^*2*^ = 0.25, *p* = 0.003), Absence of Hostility (*R* ^*2*^ = 0.28, *p* = 0.001), and Quality of Attachment (*R* ^*2*^ = 0.34, *p* < 0.001). Maternal postpartum depressive symptoms were significantly negatively associated with both Absence of Hostility (*B* = −0.20, *p* = 0.004) and Quality of Attachment (*B* = −0.54, *p* < 0.001), and paternal ADHD was significantly associated with greater maternal hostility (*B* = −1.85, *p* = 0.025). Neither postpartum depressive symptoms nor parental ADHD were associated with Pleasure in Interaction; only increased home chaos was a significant negative predictor for this subscale (*B* = −0.16, *p* = 0.004).

In the secondary analysis, inclusion of antenatal MIA as a predictor resulted in a model explaining 50% of the variance in overall postnatal MIA (*p* < 0.001). Antenatal MIA was positively associated with overall postnatal MIA (*B* = 0.65, *p* = 0.002) and with each of the three subscale outcomes (full results presented in Table S1). Postpartum depressive symptoms remained significantly associated with increased maternal hostility toward the infant (*B* = −0.15, *p* = 0.048) and decreased quality of MIA (*B =* −0.43, *p* = 0.007), but not Pleasure in Interaction with the child. As in previous analyses, maternal hostility was the sole outcome variable predicted by paternal ADHD (*B =* −2.16, *p* = 0.041).

## DISCUSSION

This study examined the effects of psychiatric and environmental variables on early MIA with the goal of clarifying the roles of parental ADHD and maternal postpartum depressive symptoms. Mean postpartum MIA for the overall sample (82.74) was on the high end of the total possible score of 95, similar to the results of prior studies by Condon and Corkindale (1998) and Le Bas et al. (2022). Findings are consistent with previously identified correlates of MIA, including postpartum depressive symptoms, history of maternal mood or anxiety disorder, and antenatal MIA, and further suggest that mothers with ADHD or coparenting with fathers with ADHD are predisposed to affective risk factors and to poorer MIA outcomes during the neonatal period. While several authors have investigated parameters shaping MIA, the present study is the first to explore the influence of parental ADHD on early maternal bonding, both generally and in relation to the sex of the parent diagnosed with ADHD.

The bivariate correlation between MIA and postpartum depressive symptoms (Dubber et al., 2015; Makeen et al., 2022) and history of prior maternal mood or anxiety disorder (Farré‐Sender et al., 2018; Petri et al., 2018) in our sample echoed previous literature assessing MIA between one and four months postpartum. The inverse relationship between MIA and postpartum depressive symptoms found here was robust to adjustment for demographic covariates, participant ADHD, and other elements of the parenting environment. Interestingly, negative associations at one month postpartum between depressive symptoms and Quality of Attachment and Absence of Hostility (but not Pleasure in Interaction) subscales reflected findings at six to eight months postpartum by Behrendt et al.‘s 2016 study of primiparous mothers with and without subclinical depression, suggesting enjoyment of parent‐child interaction may be less affected by parental mood symptoms than other aspects of attachment.

As hypothesized, mothers in families with parental ADHD (either self‐ or coparent) reported significantly more postpartum depressive symptoms than those in neurotypical parent dyads. Specifically, compared to mothers coparenting with fathers with ADHD, and to those in couples without ADHD, mothers diagnosed with ADHD endorsed higher postpartum depressive symptoms and home chaos, factors significantly associated with poorer MIA in our sample. Previous work has shown ADHD symptoms to be positively correlated with rumination, a cognitive distortion commonly associated with major depression that has also been linked to dysfunctional mother‐infant interaction (Fredrick et al., 2020; Kandeğer et al., 2023). Findings in the present study align with prior research demonstrating mothers with ADHD or with high polygenic risk scores for ADHD are more likely to develop postpartum depression and anxiety in the first year of infant life (Andersson et al., 2023), experience more household chaos (Agnew‐Blais et al., 2022), and have greater sleep disturbances (Joseph et al., 2022). Despite this, ANOVA comparisons revealed that while MIA was highest among mothers in neurotypical parenting pairs, mothers diagnosed with ADHD reported stronger MIA than those with fathers with ADHD. Likewise, stepwise regression analyses showed that the negative association between parental ADHD and MIA was driven primarily by paternal ADHD in our sample.

One theoretical link between coparent ADHD and impaired MIA is greater coparenting stress related to inequitable distribution of parenting/household responsibilities. Eakin et al. (2004) found that spouses of ADHD adults had poorer marital satisfaction than controls, with over 90% reporting compensation for their spouses in household organization, time management, and parenting activities. During the stressful transition to parenthood, a significant rise in compensatory demands on the unaffected partner of an ADHD coparent may negatively affect parent‐infant bonding. This hypothesis is supported by recent findings from Joseph et al. (2022), who reported that adults with parental ADHD (self‐ or coparent) experienced greater parenting distress in the first year of life than those without. Evidence indicates social support is highly protective against postpartum depression (Pao et al., 2019) and is also associated with improved peripartum MIA (Delavari et al., 2018; Hopkins et al., 2018). While in the present study, maternal ratings of social support did not correlate with postpartum MIA, social support is a multidimensional variable capturing several aspects of socioeconomic security and community and family engagement. Of these, the coparent relationship may represent the key factor for MIA; Bain et al. (2024) noted that while positive spousal relationships and social support both strengthened maternal‐infant responsiveness, among depressed women, spousal support was especially critical. The general social support measure utilized in this analysis may have insufficiently described the coparent relationship, particularly regarding ADHD‐related dysfunction, to reveal a relationship between social support and MIA.

Regression results suggest that coparent ADHD was more strongly associated with increased maternal hostility toward the infant than other aspects of MIA. While there is a paucity of literature exploring parental ADHD and infant‐related hostility, findings by Bertrand et al. (2024) may offer insight into one potential explanation. In their study of mothers of young children (3–7 years), they observed greater hostile attribution bias toward their children when maternal executive functioning was low. Thus, difficulty with planning, organizing, and other higher‐order cognitive skills involved in parenting may incite frustration when children increase demands on limited cognitive resources. Similarly, compensation for coparent ADHD may strain executive functioning capacity when help from the other parent is limited, resulting in increased parental hostility toward the child. Although not examined here, if mothers coparenting with a father who has ADHD assume more of the childcare responsibilities (e.g., changing and feeding the infant), this may lead to feelings of resentment and increased hostility associated with caregiving. Regardless of parental ADHD status, postpartum depressive and anxiety symptoms are known to impair executive functioning (Warren et al., 2021) and thus may also contribute to increased parental hostility toward infants. In support of this possibility, maternal postpartum depressive symptoms were related to maternal hostility alongside father ADHD, suggesting that these correlated risk factors for maternal hostility to the child may track together and worsen MIA.

Another possibility is that infants with parental ADHD who are at high risk of developing childhood ADHD (Faraone & Larsson, 2019), have more difficult temperaments, contributing to strained MIA in families with parental ADHD. In a meta‐analysis, greater negative emotionality, increased activity level, and lower sustained attention in infancy and toddlerhood have been found to prospectively predict later development of childhood ADHD (Joseph et al., 2023). Future work should examine the relations between infant temperament, parental psychopathology (e.g. depression, ADHD), and parent‐child attachment.

Although this study identified a statistically significant relationship between parental ADHD and early MIA, replication of these preliminary results in a larger population is essential. Lack of prior research on the impact of parental ADHD on MIA limited our ability to conduct an accurate power analysis, however, small sample size likely reduced power to detect effects smaller than moderate. Nonetheless, several factors support the credibility of our findings, including plausible pathways linking parental ADHD to early parent‐child attachment (e.g. increased risk of lifetime and postpartum depression for parents with ADHD, greater executive functioning demands in early parenthood, and more difficult infant temperament among children later diagnosed with ADHD) and consistency with the literature on the deleterious contribution of postpartum depressive symptoms to MIA. Additionally, prospective study design and use of standardized clinical interviews to validate ADHD and other lifetime psychiatric conditions ensured both diagnostic accuracy and appropriate temporality of exposure to stable predictor variables, including parent demographics, ADHD status, health histories, and parenting environment factors.

Moreover, the present study consistently found statistically significant associations between maternal ADHD, paternal ADHD, and diminished MIA across correlational analyses, analysis of variance tests, and regression models adjusted for maternal characteristics and known predictors of poor MIA (i.e. depressive symptoms). Given the statistical significance of ADHD as a correlate of MIA in both family‐level (either parent) and gendered parental ADHD regression models, the marginal significance of paternal ADHD in the final model is likely due to the small subsample of participants coparenting with fathers with ADHD (*n* = 13). Future research should not only incorporate maternal self‐report measures of attachment (as presented in this study) but also integrate observational data on parent‐child interactions to characterize the early mother‐child relationship more fully. Additionally, although we were unable to examine the impact of prenatal psychiatric medication on MIA due to the small number of mothers in our sample who reported using ADHD medications (*n* = 5) during pregnancy or postpartum, further research is needed to determine whether pharmacologic interventions influence the relationship between maternal mental health symptoms and MIA.

Finally, the generalizability of this study is limited by the demographic homogeneity of its primarily white, highly educated sample. Because the New(born) PARIS study aimed to identify infants at increased genetic risk of ADHD, only biological parent pairs were enrolled. While gender identity was not an inclusion criterion, all enrolled parents were cisgender and parenting in heterosexual relationships. As a result, our findings may be less applicable to gender diverse parents or those in non‐heterosexual pairings, who may experience different psychosocial stressors and parenting dynamics than participants in our sample.

## CONCLUSIONS

Parental ADHD is associated with increased postpartum depressive symptoms and greater home chaos and may interfere with early MIA. Existing interventions for strengthening MIA primarily target mothers with obstetric complications or postpartum depression (Mascheroni & Ionio, 2019), and none have yet targeted parental ADHD. Results suggest that preventative interventions aimed at improving ADHD symptoms and related impairments among expectant families with parental ADHD may enhance maternal bonding, healthy family functioning, and long‐term mental health outcomes for mothers and their children.

## AUTHOR CONTRIBUTIONS

**Elyse Mark**: Conceptualization; formal analysis; methodology; writing—original draft; writing—review and editing. **Brooke S. G. Molina**: Supervision; writing—review and editing. **Michelle A. Wilson**: Data curation; project administration. **Charles H. Zeanah**: Supervision; writing—review and editing. **Heather M. Joseph**: Conceptualization; funding acquisition; methodology; resources; supervision; writing— review and editing.

## CONFLICT OF INTEREST STATEMENT

The authors declare no conflicts of interest.

## ETHICAL CONSIDERATIONS

This study has been reviewed and approved by the University of Pittsburgh Institutional Review Board (STUDY21080047). Written informed consent was obtained from participants before research activities were conducted for this study.

## Supporting information

Table S1

## Data Availability

The data that support the findings of this study are available from the corresponding author upon reasonable request.

## ENDNOTE

^1^
Lifetime history of Major Depressive Disorder, Bipolar I or II disorder, Generalized Anxiety Disorder, Panic Disorder, or Social Anxiety Disorder, operationalized as a binary variable
