# Supplementary material for: Differential impacts of parental attention deficit/hyperactivity disorder on early maternal‐infant attachment
Source: JCPP Adv. 2025 Jun 27;6(1):e70029. doi: 10.1002/jcv2.70029 (PMC12973118; doi:10.1002/jcv2.70029)
Supplement: Supplementary file 1 — Table S1 [file JCV2-6-e70029-s001.docx]

**Table S1.** Predictive value of antenatal MIA on postnatal MIA (*n*=45)

|  |  | | MPAS subscales | | | | | |
| --- | --- | --- | --- | --- | --- | --- | --- | --- |
|  | MPAS Total Score  R^2^=0.502 (*p*<.001) | | Pleasure in Interaction  R^2^=0.249 (*p*=.015) | | Absence of Hostility  R^2^=.561 (*p*<.001) | | Quality of Attachment  R^2^=.465 (*p*=.002) | |
|  | B (SE) | *p* | B (SE) | *p* | B (SE) | *p* | B (SE) | *p* |
| Maternal age | -.05 (.37) | .885 | .04 (.13) | .769 | -.06 (.10) | .524 | -.03 (.20) | .888 |
| Parental education | -.24 (1.19) | .843 | -.07 (.43) | .875 | -.59 (.32) | .075 | .42 (.67) | .529 |
| MAAS | **.65 (.19)**** | **.002** | **.17 (.07)*** | **.019** | **.11 (.05)*** | **.040** | **.37 (.11)** | **.002** |
| Mother ADHD | -.28 (2.99) | .926 | 1.26 (1.09) | .255 | -.43 (.81) | .597 | -1.11 (1.67) | .512 |
| Father ADHD | .09 (3.75) | .981 | 1.09 (1.37) | .430 | **-2.16 (1.02)*** | **.041** | 1.16 (2.10) | .584 |
| Mother postpartum depressive symptoms | **-.60 (.27)*** | **.032** | -.02 (.10) | .855 | **-.15 (.07)*** | **.048** | **-.43 (.15)**** | **.007** |
| Mother lifetime mood disorder | -3.48 (2.50) | .171 | -1.53 (.91) | .102 | -1.10 (.68) | .112 | -.85 (1.40) | .546 |
| Home chaos | -.13 (.25) | .602 | -.18 (.09) | .206 | -.07 (.07) | .318 | .12 (.14) | .415 |
| Constant | 46.59 (26.10) | .083 | 12.25 (9.52) | .206 | 22.54 (7.08)** | .003 | 11.81 (14.61) | .424 |

*Note: *p*<.05, ***p*<.01,****p*<.001
